# Supplementary material for: Phylogenomics Provides New Insights into Gains and Losses of Selenoproteins among Archaeplastida
Source: Int J Mol Sci. 2019 Jun 20;20(12):3020. doi: 10.3390/ijms20123020 (PMC6627142; doi:10.3390/ijms20123020)
Supplement: Supplementary file 1 [file ijms-20-03020-s001.zip › Supplementary Materials.pdf]

Supplementary Materials:

Phylogenomics provides new insights into gains and losses of selenoproteins among Archaeplastida

Hongping Liang[1,2,3#], Tong Wei[2,3,4#], Yan Xu[1,2,3#], Linzhou Li[2,3,6], Sunil Kumar Sahu[2,3,4], Hongli Wang[1,2,3], Haoyuan Li[1,2], Xian Fu[2,3], Gengyun Zhang[2,4], Michael Melkonian[7], Xin Liu[2,3,4], Sibo Wang[2,4,5*],Huan Liu[2,4,5*]

[1] BGI Education Center, University of Chinese Academy of Sciences, Beijing, China.
[2] BGI-Shenzhen, Beishan Industrial Zone, Yantian District, Shenzhen 518083, China.
[3] China National Gene Bank, Institute of New Agricultural Resources, BGI-Shenzhen, Jinsha Road, Shenzhen 518120, China.
[4] State Key Laboratory of Agricultural Genomics, BGI-Shenzhen, Shenzhen 518083, China.
[5] Department of Biology, University of Copenhagen, Copenhagen, Denmark.
[6] School of Biology and Biological Engineering, South China University of Technology, 510006, China.
[7] Botanical Institute, Cologne Biocenter, University of Cologne, Cologne D-50674, Germany.
[#]these authors contributed equally to this work
* Correspondence: *liuhuan@genomics.cn

Supplementary Figure S1: Completeness of genome assemblies and Stop codon statistics. The genome quality was assessed by the BUSCO program. The number of single, multiple, missing and fragmented genes are shown in the middle histogram. The stop codon usage of genes is shown on the right histograms. The species that lost the Sec machinery are marked with an asterisk in the tree. Each group was colored in different background in the left column.

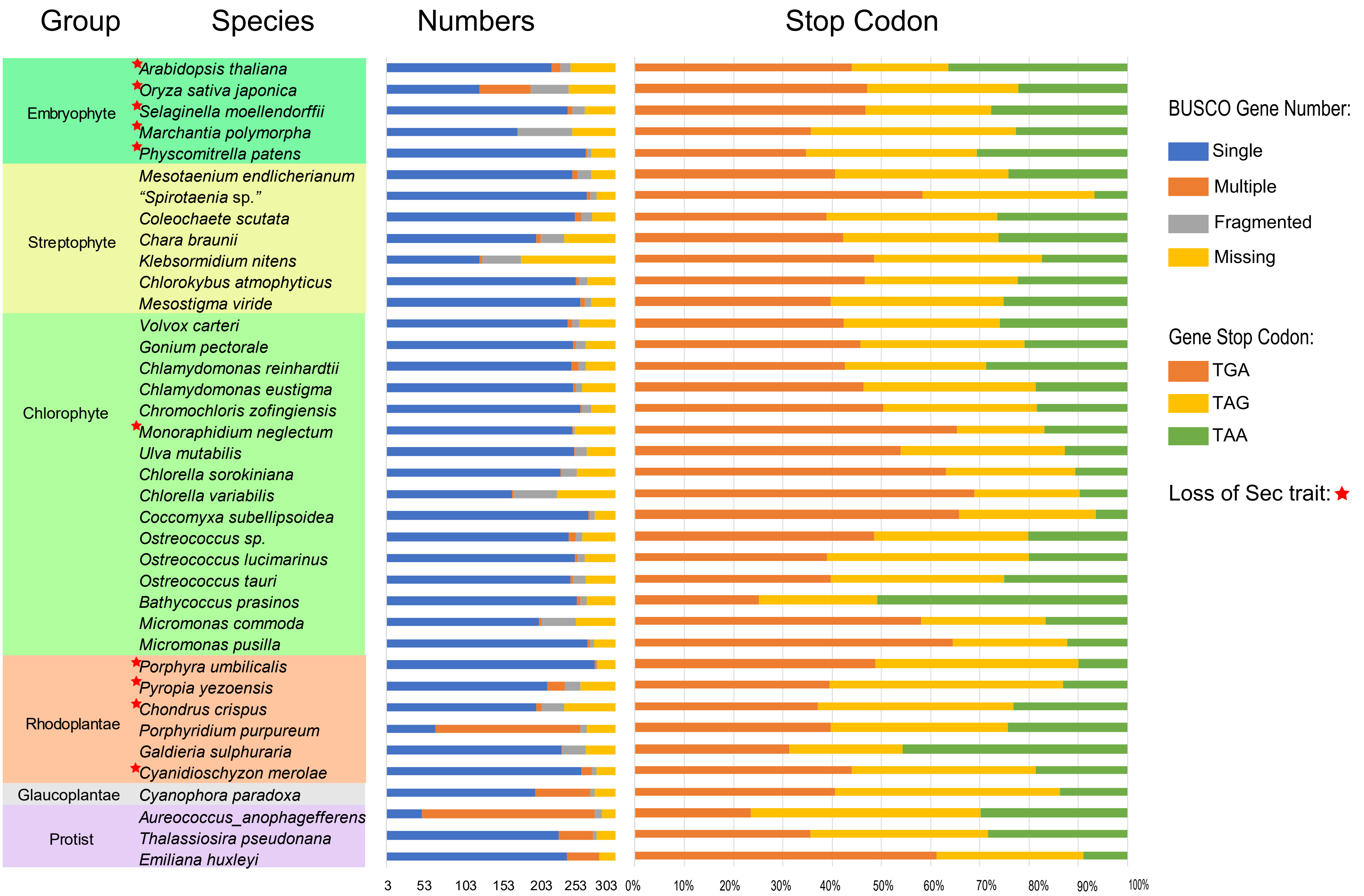

Supplementary Figure S2: Species tree with families of selenoproteins and homologues (Cys) by Selenoprofiles. Each column corresponds to a selenoprotein family plotted in different color to distinguish Sec-containing (green), Cys homologues (red), and others (grey). 33 out of 62 selenoproteins families having at least one Sec -containing gene were selected for the analysis. The number in the left box represents the number of selenoproteins.

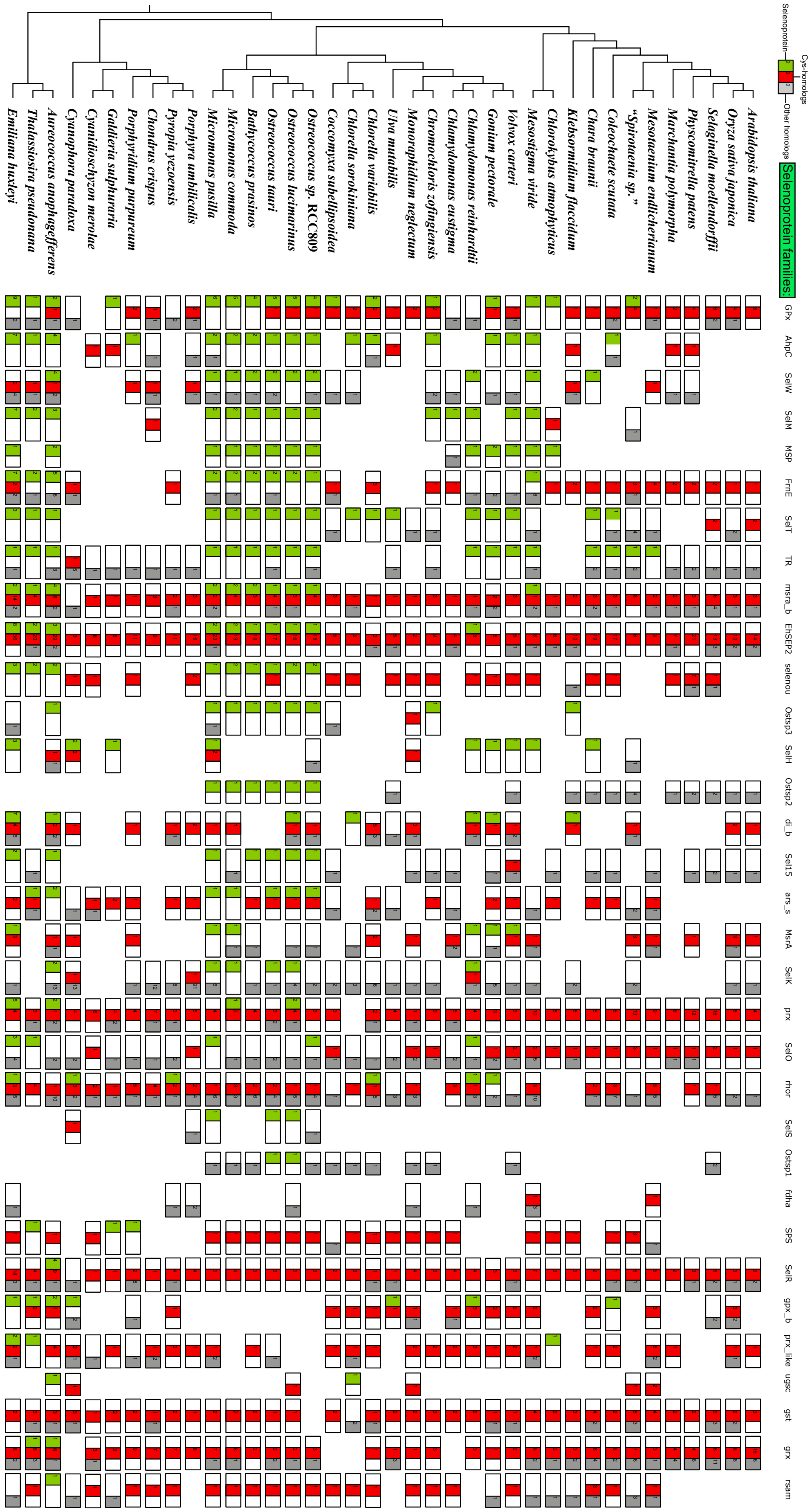

Supplementary Figure S3: The phylogenetic tree of SBP2, PSTK and SPS. All the predicted candidates were used to reconstruct a maximum-likelihood tree, with 500 bootstrap replicates. Here, Orange: Mamiellophyceae; Yellow: core Chlorophyta; Green: Streptophyte algae; Red: Rhodoplantae; and Grey: Glaucoplantae.

a. SBP2

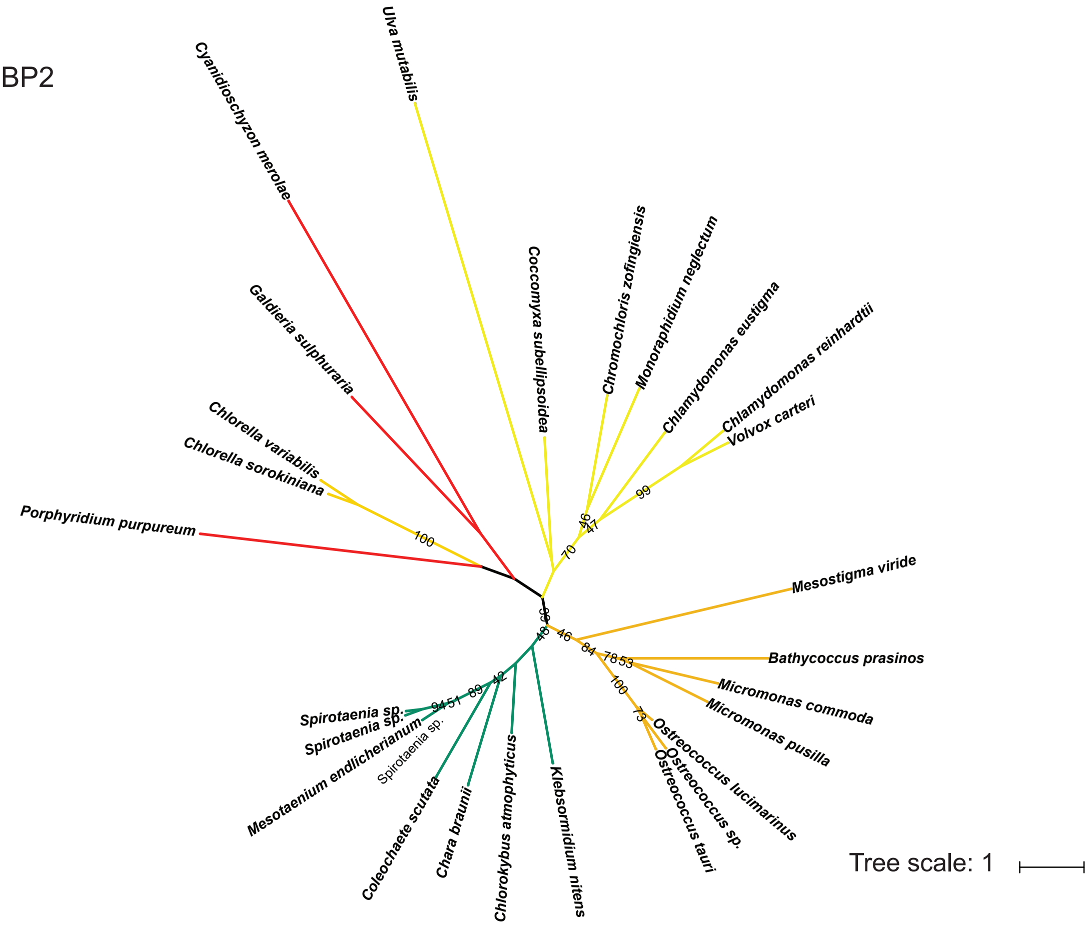

b. PSTK

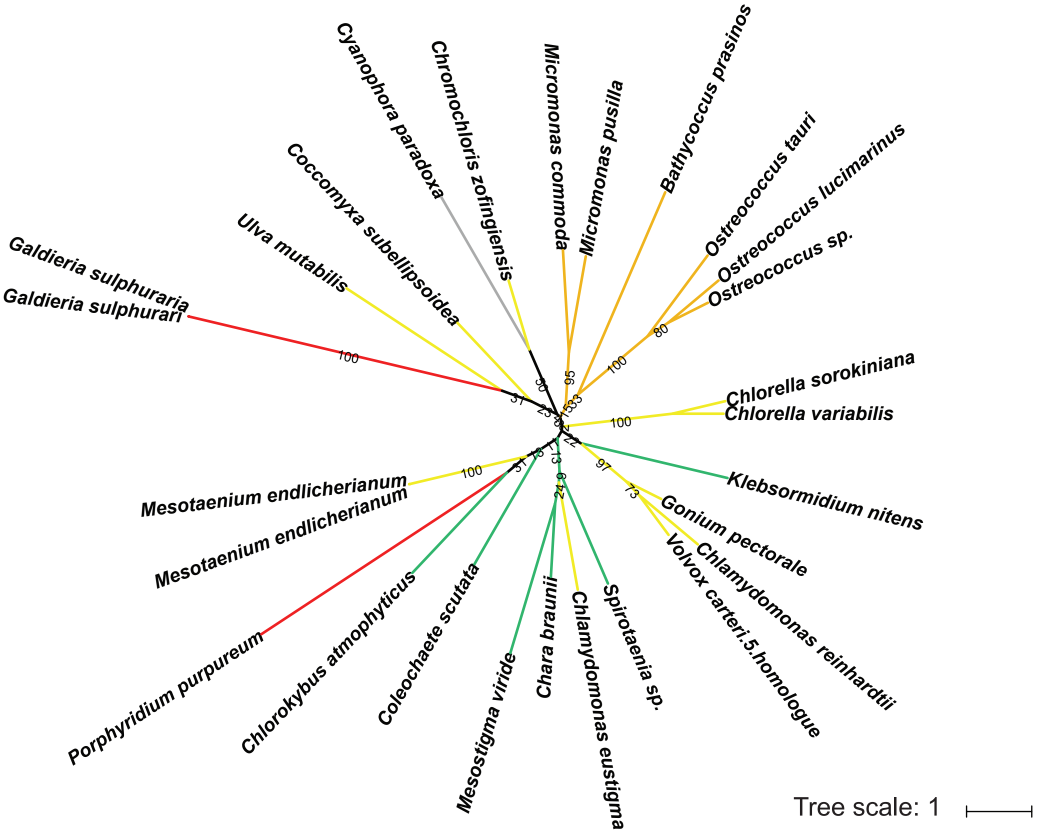

c. SPS

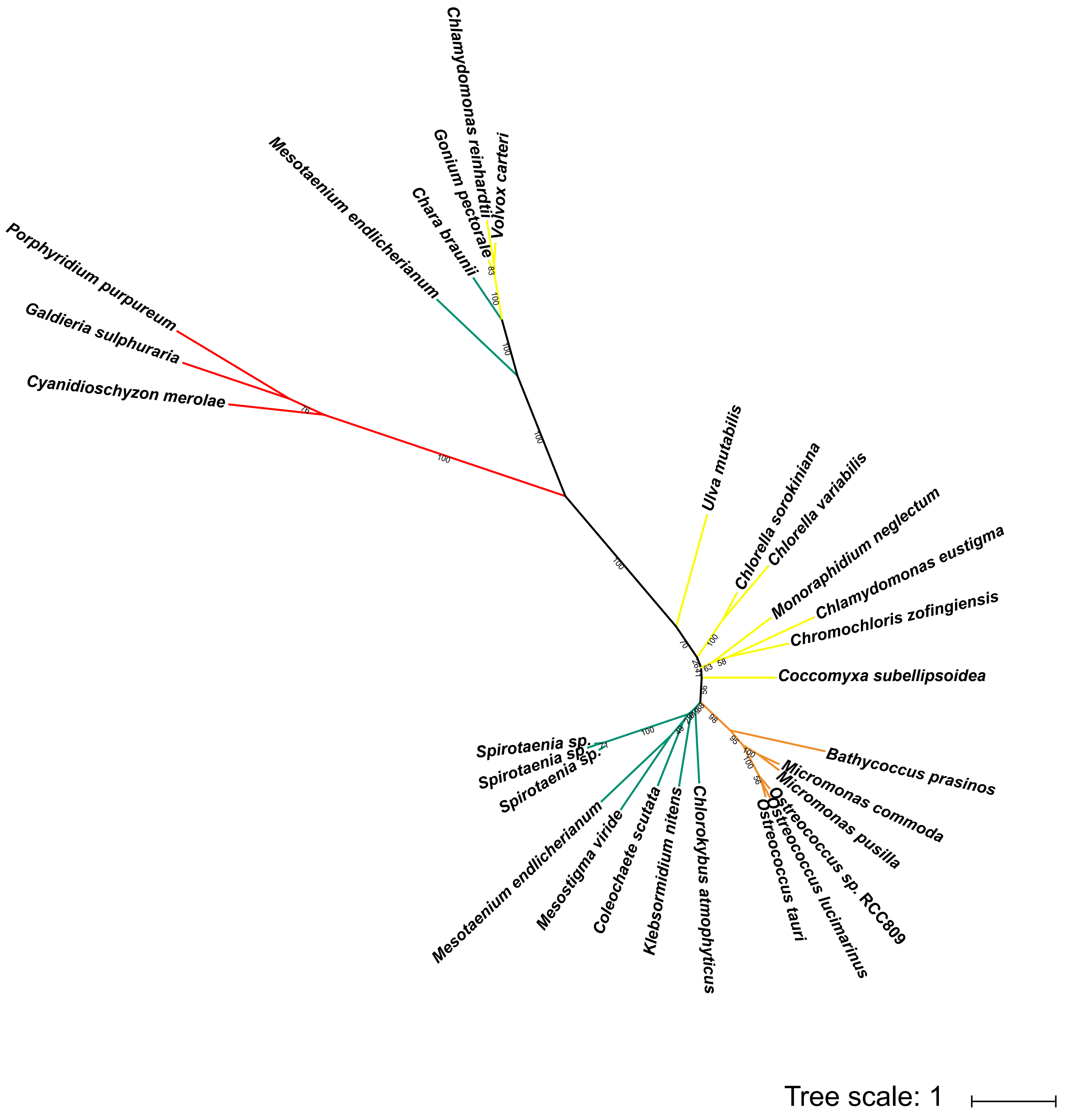
Chlamydomonas reinhardtii
Volvox carteri
Gonium pectorale
Chara braunii
Mesotaenium endlicherianum
Porphyridium purpureum
Galdieria sulphuraria
Cyanidioschyzon merolae
Ulva mutabilis
Chlorella sorokiniana
Chlorella variabilis
Monoraphidium neglectum
Chlamydomonas eustigma
Chromochloris zofingiensis
Coccomyxa subellipsoidea
Bathycoccus prasinos
Micromonas commoda
Micromonas pusilla
Ostreococcus sp. RCC809
Ostreococcus lucimarinus
Ostreococcus tauri
Chlorokybus atmophyticus
Klebsormidium nitens
Coleochaete scutata
Mesostigma viride
Mesotaenium endlicherianum
Spirotaenia sp.
Spirotaenia sp.
Spirotaenia sp.
Tree scale: 1

Supplementary Figure S4: The phylogenetic tree of SPS. The maximum-likelihood tree by using the reference set of SPS proteins was reconstructed using the GTR+I+G model.

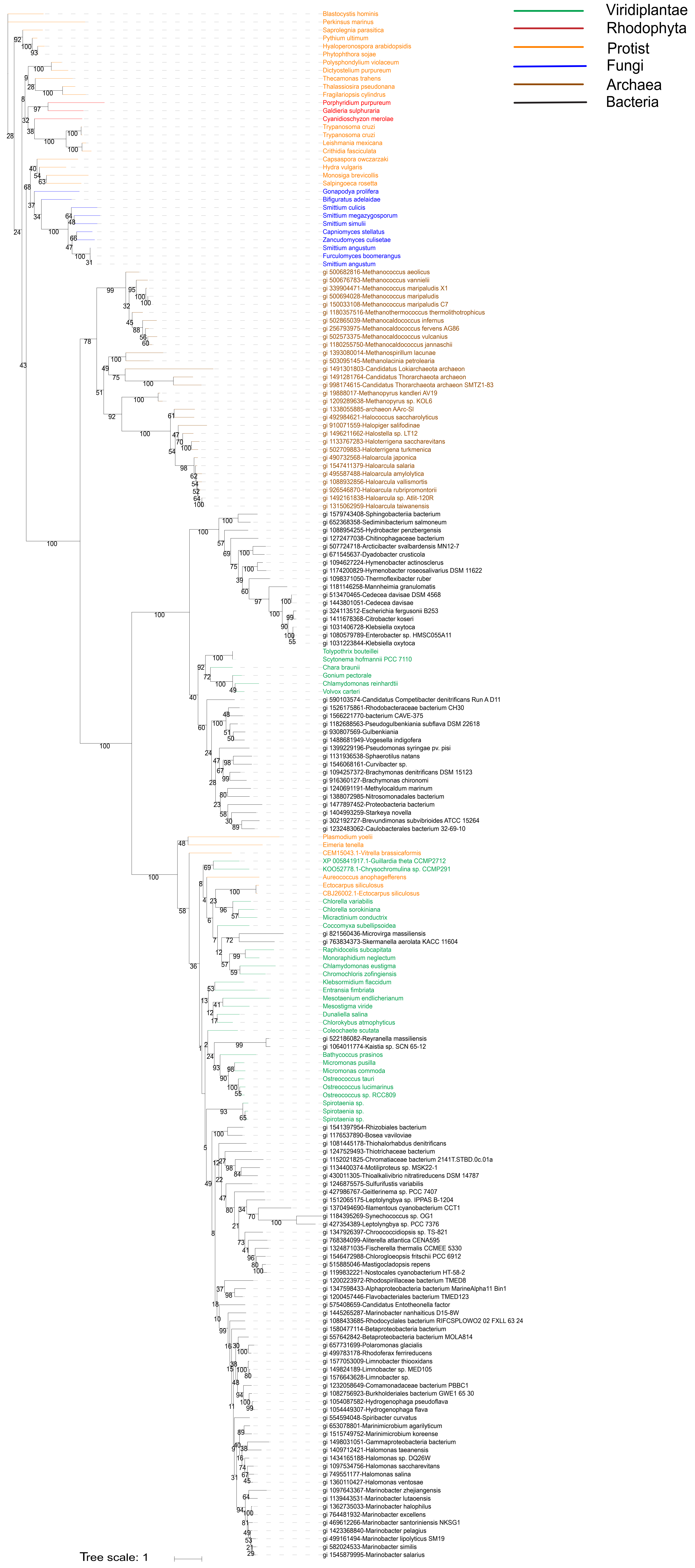

Supplementary Figure S5: The alignment of the SPS domain. The complement alignment of SPS domains in the three clades. Nucleotide conservation is indicated with red and yellow coloring. The three clades are separated by spaces. From top to bottom, they are clade III, clade II and clade I.

```
Methanothermococcus thermolithotrophicus   ............................................................
Methanocaldococcus fervens                 ............................................................
Methanocaldococcus vulcanius               ............................................................
Methanocaldococcus jannaschii              ............................................................
Methanocaldococcus infernus                ............................................................

Cedecea davisae                            ............................................................
Escherichia fergusonii                     ............................................................
Citrobacter koseri                         ............................................................
Mannheimia granulomatis                    ............................................................
Klebsiella oxytoca                         ............................................................

Burkholderiales bacterium                  DLVLIGGGHSHVGVLRMFAMKPEPGVRITVICTDIDTPYSGMLPGYISGHYSFDEVHIDL
Comamonadaceae bacterium                   DLVLVGGGHSHVVALRMLAMQPEPGLRITLVCTDIDTPYSGMLPGYVSGHYSFDEVHIDL
Hydrogenophaga flava                       DLVLVGGGHSHVVALRMLAMRPEPGLRITMVCTDIDTPYSGMLPGYIAGHYSFDEVHIDL
Hydrogenophaga pseudoflava                 DIVLVGGGHSHVGVLRMFGMKPWPGVRLTLICTDIHTPYSGMLPGYIAGHYSYDDVHIDL
Limnobacter sp.                            DLVLVGGGHSHVVVLRMLAMQPEPGLRITLICTDIDTPYSGMLPGYISGHYSFDDVHIDL


Methanothermococcus thermolithotrophicus   ............................................................
Methanocaldococcus fervens                 ............................................................
Methanocaldococcus vulcanius               ............................................................
Methanocaldococcus jannaschii              ............................................................
Methanocaldococcus infernus                ............................................................

Cedecea davisae                            ............................................................
Escherichia fergusonii                     ............................................................
Citrobacter koseri                         ............................................................
Mannheimia granulomatis                    ............................................................
Klebsiella oxytoca                         ............................................................

Burkholderiales bacterium                  GRLCAYTGARFFHDAVVGIDRQNQKVICQNRPPVAYDLLSINIGSTPQVRHVAGAQSLAV
Comamonadaceae bacterium                   GRLAAFAGARFIHGEVTGLDRASQRVLLKGRPAIPYDLLSINTGSTPNVRQVDGAQAHAV
Hydrogenophaga flava                       GRLAVFAGARFIHGEVTGLDRANQRVLLKGRPSIPYDLLSINTGSTPNVRQVDGAQAHAV
Hydrogenophaga pseudoflava                 GRLCAFAGARLFKDEVVGIDRANQKVICKNRPPVAYDALSINIGSTPQVQLVPGALENAV
Limnobacter sp.                            GRLAAFAGARFIHGEVTGLDRANQRVLLRDRPSVPYDLLSINTGSTPNVRQVDGARAHTV


Methanothermococcus thermolithotrophicus   ............................................................
Methanocaldococcus fervens                 ............................................................
Methanocaldococcus vulcanius               ............................................................
Methanocaldococcus jannaschii              ............................................................
Methanocaldococcus infernus                ............................................................

Cedecea davisae                            ............................................................
Escherichia fergusonii                     ............................................................
Citrobacter koseri                         ............................................................
Mannheimia granulomatis                    ............................................................
Klebsiella oxytoca                         ............................................................

Burkholderiales bacterium                  PVKPIAQFNQRWLALLDKARQWPIHRGRMTIAVVGAGAGGVELVLSMQYRLRNELKALGR
Comamonadaceae bacterium                   PVKPIAHFNQRWLDLLERVRGL...RDRFTVAVVGGGAGGVELVLSMQYRLRQELQALGK
Hydrogenophaga flava                       PVKPIAHFNQRWLALLERVRSL...RDRFTVAVVGGGAGGVELVLSMQYRLRQELQALGK
Hydrogenophaga pseudoflava                 PVKPIARFNQRWVNLLDRVKTH...AGKTTIAVVGGGAGGVELALAMQFRLRNELSTMGR
Limnobacter sp.                            PVKPIAHFNQRWLQLLDRVRGL...HSRFTIAVVGGGAGGVELVLSVQFRLRNELLKLGR


Methanothermococcus thermolithotrophicus   ............................................................
Methanocaldococcus fervens                 ............................................................
Methanocaldococcus vulcanius               ............................................................
Methanocaldococcus jannaschii              ............................................................
Methanocaldococcus infernus                ............................................................

Cedecea davisae                            ............................................................
Escherichia fergusonii                     ............................................................
Citrobacter koseri                         ............................................................
Mannheimia granulomatis                    ............................................................
Klebsiella oxytoca                         ............................................................

Burkholderiales bacterium                  NPEDLQFVLLTAGETILPTHNPGVRARFARVLQARNVTVHTQTEVVEVSPGCLHTRDGRT
Comamonadaceae bacterium                   NPDLLQFVLLTAGETLLPTHNPRVRARFAHVLKERRVAVHTRAEVSQVSPGCLTTQDGRT
Hydrogenophaga flava                       SPDLLQFVLLTAGDSLLPTHNPRVRARFAHVLKERRVAVHTRAEVTQVSPGCLTTQDGRT
Hydrogenophaga pseudoflava                 NPDELEFHLFTADADVLPTHNPGVRARFEKVLNERGVVLHRDAEVVQLDGHTLQTKQGER
Limnobacter sp.                            NPDLLRFVLLTAGDTILPTHNPGVRARFARVLKERHVAVHTRAEVTEVSPGCLHTQDGRT


                                                                                                   1
Methanothermococcus thermolithotrophicus   ........................................................DDAA
Methanocaldococcus fervens                 ........................................................DDAA
Methanocaldococcus vulcanius               ........................................................DDAA
Methanocaldococcus jannaschii              ........................................................DDAS
Methanocaldococcus infernus                ........................................................DDAS

Cedecea davisae                            ........................................................DDAA
Escherichia fergusonii                     ........................................................DDAA
Citrobacter koseri                         ........................................................DDAA
Mannheimia granulomatis                    ........................................................DDAA
Klebsiella oxytoca                         ........................................................DDAA

Burkholderiales bacterium                  FDADDTLWVTQAGGPAWLKSTGLALDKQGFILVHPQLQTLNDPLVFAAGDIA....DDAA
Comamonadaceae bacterium                   FDADETMWVTQAGGPAWLQGTGLALDEHGFVCVNEYLQTPDDPKIYAAGDVASFLPDDAA
Hydrogenophaga flava                       FDADETMWVTQAGGPAWLQGTGLALDEHGFVCVNEYLQTPDDPKIFAAGDVASFLPDDAA
Hydrogenophaga pseudoflava                 LHADEIMWVTQAGGAAWLKNTDLELDSRGFINVGPTLQTTRDAKIFAAGDIAN...DDAA
Limnobacter sp.                            FDADETLWVTQAGGPVWLQSTGLALDEHGFIQVNQQLQTLDDPKIFAVGDVA....DDAA
```

```
                                                170       180       190       200       210       220
Methanothermococcus thermolithotrophicus  DILTKAGAKEGDVLILTKPLGTQTAMALSRVTEEFEDLLDIEKPEKEYIINKAIELMTTS
Methanocaldococcus fervens                EVLTKAGAKEGDILILTKPLGTQTAMALSRIPEEFKDLIDVSEEEKEYIINKAIELMTTS
Methanocaldococcus vulcanius              EVLTKAGAEEGDVLILTKPLGTQTAMALSRIPDDYKELIGIDEEEMNYIINKAIELMTTS
Methanocaldococcus jannaschii             EVLTKAGVKVGDVLILTKPLGTQTAMALSRIPEEFKDLISITEEERDYIINKAIEIMTTS
Methanocaldococcus infernus               EVLTKAGAKVGDVLILTKPLGTQSAMALSRVPKEFLELINISEEEKEEIINKAIKLMTTS

Cedecea davisae                           RVKKNSSAEAGCKLFLTKPLGIGVLTTAEK...........KSLLLPEHQGLAAETMCQL
Escherichia fergusonii                    RVKKNSTAQAGCKLFLTKPLGIGVLTTAEK...........KSLLKPEHQGLATEVMCRM
Citrobacter koseri                        RVKKNSTAQAGCKLFLTKPLGIGVLTTAEK...........KSLLKPEHQGLATEVMCRM
Mannheimia granulomatis                   RVKRNASAVAGCELFLTKPLGIGILTTAEK...........KGLLQPEHQHLASEVMCQI
Klebsiella oxytoca                        RVKKNSTAQAGCKLYLTKPLGIGVLTTAEK...........KSLLKPEHQGLATETMCQM

Burkholderiales bacterium                 GVMRKGGMLPGDVLLLTKPIGTGTLFAAHA...........RYAAKGRWIDAALKSMVVS
Comamonadaceae bacterium                  SVMRKGGMQPGDVLLLTKPIGTGTLFAAHA...........RYAAKGRWIDAALQSMVLS
Hydrogenophaga flava                      GVMRKGGMRPGDVLLLTKPIGTGTLFAAHA...........QHAAKGRWVDATLQSMVVS
Hydrogenophaga pseudoflava                GVMRKGGMRPGDVLLLSKPIGTGTLFAAHA...........RHAAKGRWIDAALKSMVLS
Limnobacter sp.                           GVMIKGGMRPGDAILLTKPIGTGTLFAALP...........QLKTKGRWIDAALESMVKS

                                                230       240       250       260       270         280
Methanothermococcus thermolithotrophicus  NRKALLILRKLEEGLNEKVANAMTDITGFGLLGHSNEMAEQSNVEIEINR..LPLIKNTG
Methanocaldococcus fervens                NRYALMALRRAEKRVGDKIANALTDITGFGILGHSNEMADNSNVLIEINL..LPCIKKTP
Methanocaldococcus vulcanius              NRYALKSLRNAEKKVGDKVANALTDITGFGLLGHSNEMAKNSNVLIEINL..LPCIKRTP
Methanocaldococcus jannaschii             NRYALKALRKAEERVGDKIANALTDITGFGILGHSNEMAKNSNVLIEINL..LPCIKRTP
Methanocaldococcus infernus               NKYSLEALRRAEEKVGDKIANALTDVTGFGLLGHSNEIAKQSKVNIKIDT..LPCIKKTP

Cedecea davisae                           NKVGTEFADIAG.......VTAMTDVTGFGLLGHLSEMCQGAGVQADVWFECVPKLPGVE
Escherichia fergusonii                    NIAGASFANIEG.......VKAMTDVTGFGLLGHLSEMCQGAGVQARVDYDAIPKLPGVE
Citrobacter koseri                        NIAGASFAHIDG.......VKAMTDVTGFGLLGHLSEMCQGAGVQARICYQDIPKLPGVE
Mannheimia granulomatis                   NLIGAEFSELPE.......VTAMTDVTGFGLLGHLSEVCQGSNVRAEVHFADIQTLDGTK
Klebsiella oxytoca                        NLVGSAFANIDG.......VKAMTDVTGFGLLGHLSEVCRGAGVQAQLTYASIPKLPGVE

Burkholderiales bacterium                 NQAGAQILRAHG.......ATACTDLTGFGLLGHLVEMTRPSGVDAELQLSALPLLDGAV
Comamonadaceae bacterium                  NQSGAQILRTHG.......ATACTDLTGFGLLGHLVEMTRPSGVDAELQLSTLPLLDGAV
Hydrogenophaga flava                      NQAGARILREHG.......ATACTDLTGFGLLGHLVEMTRPSGMDAELQLGALPLLDGAV
Hydrogenophaga pseudoflava                NQAGAQILREHG.......ATACTDLTGFGLLGHLVEMTRPSGVDAELQLGALPLLDGAV
Limnobacter sp.                           NRLGAQCLREYG.......SKACTDLTGFGLLGHLVEMTRPSEVDAELDLSALPLLDGAL

                                                               290                   300       310
Methanothermococcus thermolithotrophicus  RLSSM.............FGHSLLKG............TGAETAGGLLISVKKQYKDALI
Methanocaldococcus fervens                ELSRL.............FGHALLDG............YGAETAGGLLISAKKEYKDDLI
Methanocaldococcus vulcanius              ELSKM.............FGHALLDG............YGAETAGGLLISTKNDYKDDLI
Methanocaldococcus jannaschii             ELSRL.............FGHALLDG............YGAETAGGLLISAKEEYKDNLI
Methanocaldococcus infernus               ELSKM.............FGHALLEG............YGAETAGGLLIVAKREYKDELI

Cedecea davisae                           SYIEQGCVPGGTSRNFASYGQLVGDMP....EAWRNLLCDPQTSGGLLLAVRPDAEADVF
Escherichia fergusonii                    EYIKLGAVPGGTERNFASYGHLMGEMP....REVRDLLCDPQTSGGLLLAVTPEAEDEVK
Citrobacter koseri                        EYIKLGAVPGGTERNFASYGHLMGDMP....REVRDLLCDPQTSGGLLLAVTPEAVEEVK
Mannheimia granulomatis                   DYIALGAVPGGTTRNFDSYGHLISAMT....DEQKAILCDPQTSGGLLVAVLPQAVEKVQ
Klebsiella oxytoca                        EYIAAGAVPGGTGRNFASYGHLMGEMP....PEWRDLLCDPQTSGGLLLAVTPEAEDEVL

Burkholderiales bacterium                 ECVEAGIVSSLQPANVR.LRRALRNAEDFVKDPRYPLLFDPQTAGGLLASVPADRATDCI
Comamonadaceae bacterium                  DCVQAGIVSSLQPANVR.LRRALRNGDEFVGDPRYPLLFDPQTAGGLLASVPAAQADACV
Hydrogenophaga flava                      ECVQAGIVSSLQPANVR.LRRALRNAEAFVDDPRYPLLFDPQTAGGLLASVPADRAADCV
Hydrogenophaga pseudoflava                DCVKAGIVSSLQPANVR.LRRALRNAVAFVDDQRYPLLFDPQTAGGLLASVPADRAADCV
Limnobacter sp.                           DMVSAGIVSSLQPANVR.LRRAIRNQAEYVNDPRYPLIFDPQTAGGLLATVPGEQAEACV

                                             320        330        340       350
Methanothermococcus thermolithotrophicus  EELQKNQ.CYAFEVGTVKKSGVG.KAYLRDDVEILEVEGKAV.
Methanocaldococcus fervens                NELEKSN.CYAFEVGRVVKKGEG.KAVLSKDVKVIEIDK....
Methanocaldococcus vulcanius              EELRKNK.VYAFEVGKVVKKGEG.RAILSKDVKIVEVVGK...
Methanocaldococcus jannaschii             DELEKAK.CYAFEVGRVVKKGEG.KAVLSKDVKVIEIEGRAI.
Methanocaldococcus infernus               DELKKVG..YAFEVGEVVGRGD..KATLSKDLKIIEVEGKAV.

Cedecea davisae                           AVAERNG.IKLSPIGELKDARAG.RPMIEI......RA.....
Escherichia fergusonii                    ATAAEFG.IELSAIGELVSARGG.RAMVEI......LGGRAMV
Citrobacter koseri                        VTAAEFG.IELTAIGELVDARGG.RAMVEI......RGGRAMV
Mannheimia granulomatis                   QIAKNAC.VSLFHVGRLLEQEEG.KALIEV......IEGKA..
Klebsiella oxytoca                        AAAAEYG.ITLTAIGELVTARGG.RPMIEI......RGGR...

Burkholderiales bacterium                 RALKAAGYPQTAAIGRIRGASDVLEPVVLT......A......
Comamonadaceae bacterium                  QALKAAGYVHTAIIGRITLQGDAIEPVLLK......T......
Hydrogenophaga flava                      RALQAAGYPHTAAIGRVSAASDALEPVVLS......A......
Hydrogenophaga pseudoflava                RALQAAGYPHTAAIGRISAASDALEPVVLS......AE.....
Limnobacter sp.                           AALKKLGYEHTAIIGRILPQGEAIEPIVLK......G......
```
